# Outcome of Surgery for Unstable Intertrochanteric Fractures in Octogenarians

**DOI:** 10.5704/MOJ.1403.014

**Published:** 2014-03

**Authors:** MV Valera, L Bonifacio, SA Basman

**Affiliations:** Philippine Orthopedic Center, Maria Clara St. corner Banawe Ave., Quezon City, Philippines; Philippine Orthopedic Center, Maria Clara St. corner Banawe Ave., Quezon City, Philippines; Philippine Orthopedic Center, Maria Clara St. corner Banawe Ave., Quezon City, Philippines

## Abstract

This study aims to determine pre-morbid parameters as possible predictors of outcome of hip fracture in octogenarians with unstable intertrochanteric fracture treated operatively. Presence of co-morbidities, pre-injury level of ambulation, type of surgery, and period of delay in surgery were considered, and their effect on the post-operative outcome was evaluated using the Harris Hip Score. The computed probability of survival of octogenarians who had surgery was approximately 11 months. In patients with two or more co-morbidities, there is a significant effect on Harris Hip Score in terms of pain and deformity. Delay in surgery of more than two weeks significantly decreased the distance travelled at one year. The overall recovery is correlated to preinjury level of ambulation and delay in surgery. Patients with intertochanteric fracture in this age group, who have less co-morbidities and with more independent ambulation, are good candidates for timely operative treatment.

## Introduction

The mean age of the world's population is increasing, which is attributed to the decline in fertility and increasing life span due to improved public health measures. This global trend features increasing health care costs due to the rise in the number of people with chronic illnesses and disabilities. Although Europe and North America have the highest proportion of population aged above 65 years, the largest increase in absolute numbers of older people is predicted to occur in developing countries^1^.

In the Philippines, the population from 2010 to 2050 is projected to increase by 60%, from 93.5 million to 146 million. The population of the elderly aged 65 and above will increase almost three times from 3.9M to 17million in this period and it is also noted that the “oldest of the old” (above 80 years) is the fastest growing group^2^. The expected rise in the elderly population is also coupled with an increase in illnesses and injuries related to senescence. In one local study in 2004, using PhilHealth reimbursement claims data, the hip fracture prevalence was highest in the octogenarian group. In developing countries, hip fragility fractures impose a heavy burden to the family, relatives and community with the high cost of medical care. This creates a significant cause for concern on personal and family finances as well as public resources^3^. In the Philippine Orthopedic Center, around 78 patients were admitted in 2008 because of fractures in the intertrochanteric area. Among these, 31 (40%) patients underwent operative treatment with 17 patients undergoing compressive hip screw fixation and 14 undergoing partial hip arthroplasty. The outcomes were, however not evaluated.

In a study by Raunest of elderly patients with femoral fractures treated operatively , it was noted that a combination of polymorbidity and age beyond 78 years were significant risk factors towards morbidity and mortality after hip surgery. He also emphasized the importance of early operative treatment within a posttraumatic period of 12-24 hours^4^.

In another study by Kopp et al, patients over 70 years had a significantly shorter time of survival following the surgical treatment of proximal femoral fracture. Older age, male gender, multiple co-morbidities, poor pre-injury mobility, development of pressure sores post-operatively, failed osteosynthesis requiring revision surgery and deep infection of the affected hip were the risk factors. The fracture type, time between injury and surgery, type of anaesthesia and operative technique were found to have no significant effect to the shorter survival post-operatively^5^.

Hospital mortality after hip fracture in elderly patients was noted to be 3.2% in a study by Andress et al. Around 78 patients were studied and showed reduction of daily life activity and a reduction of Harris Hip score after operation of hip fractures^6^. Another study by Dzupa et al reported that around 35% of patients (85 out of 244) with femoral fracture died after one year of operation^7^. In a study by Holt et al, mortality at 30 and 120 days was higher in the extremely elderly (above 70 years), and those returning home were less likely to return to previous levels of mobility^8^.

We used the Harris Hip Score because it was determined to be more responsive for hip function study compared to generic measures (SF 36) for osteoarthritis of the hip^9^. This was confirmed in the studies of Shi et al^10^ and Frihagen et al^11^ on the responsiveness of Harris Hip Score on patients who underwent total hip arthroplasty and patients with femoral neck fractures.

This study focussed on a highly regarded and yet often neglected segment of our population, the octogenarians. It aimed to determine outcome predictors for hip fractures, particularly unstable intertrochanteric fractures of the femur, treated operatively in this delicate age group. Their overall survivability and functional outcome using the Harris Hip Score after the operation were investigated. Finally, this study correlated these two parameters to pre-injury factors namely, level of ambulation prior to injury, comorbidities and delay in surgery.

## Materials and Methods

### 

Forty-three patients aged 80 years and above who were admitted with a diagnosis of unstable intertrochanteric fracture at the Philippine Orthopedic Center from January 1, 2010 to December 31, 2010 were included in the study. Diagnosis of unstable intertrochanter fracture was made based on the involvement of the lesser trochanter and/or involving three or more parts fracture of the intertrochanteric area of the femur. All patients had operative treatment after initial balanced skeletal traction carried out by a number of different surgeons, residents and consultants. The decision for the choice of fixation type and surgical approach was based on the preoperative discussions between residents and consultants in the hospital.

Of the 43 patients, 28 (65%) had compression hip screw fixation (CHSF), seven (16%) dynamic condylar screw (DCS) and eight (19%) partial hip arthroplasty (PHA)

Medical records were reviewed throughout the hospital admission to collect information on comorbid medical problems and type of fracture, and aspects of medical care. Patient demographics are shown in Table I. Those patients with more than two comorbidities were categorised as high risk, and those with only one were low risk. Ambulatory status was also investigated pre and postoperatively. Patients able to ambulate without assistive devices such as canes and walkers were classified as independent ambulators. Delay of surgery from the time of injury was also noted. Patients were divided based on whether they were operated less than or more than two weeks from time of injury.

All patients with previous operation on either hip, with congenital deformity and with previous disability were excluded from the study. Patients younger than 80 years of age, multiple trauma, pathological fractures, bilateral hip fractures, reverse-obliquity type, or previous fracture or surgery on the current- fracture- site were also excluded from the study.

Information on each patient's functional status was obtained by interview using Harris Hip Score which consists of 10 subscales. Each item has different scores with pain (maximum score of 44), limp (11), support (11), distance walked (11), sitting (5), use of public transportation (1), use of stairs (4), ability to put on shoes (4), absence of deformity (4) and range of motion (4). The scores were obtained in interviews upon admission, at six weeks, at three six and 12 months post-operatively.

Survival probability of patients at any given time was analyzed using the Kaplan Meier method. For each time interval, survival probability was calculated as number of patients still alive divided by patients at risk. Patients who had dropped out were not counted (censored) as at risk. Probability of survival at any point is estimated from cumulative probability of surviving at each preceding time interval at follow up. Using the log rank test (Mantel Cox), the type of surgery, delay and comorbidities were studied to determine the significance in affecting the survival curve.

Discrimination analysis using the F-test was used to determine significant parts of the Harris Hip Score that are affected by the factors in octogenarians such as high or low risk (comorbidity), delay of surgery (less than or more than two weeks), type of surgery and ambulation (independent or dependent). Test of proportion was done to evaluate the significance of the decrease in independence of ambulation prior to surgery.

The correlation between the type of surgery, age, delay in surgery, comorbidities, ambulation and different items in Harris Hip Score were measured using the Spearman correlation coefficient. Factors which correlated were analyzed using the ANOVA to determine the trend of the results. To evaluate which part of the Harris Hip Score contributed most to the functional recovery (change of Harris Hip Score in 12 months, ambulation at 12 months) of octogenarians post-operatively, multiple linear regression with the F-test was used.

## Results

A total of 43 patients were included in the study. There were six patients (14%) who were lost to follow up. The mortality rate was 17% with a total of seven patients dying within one year. The most common cause of mortality was medical illnesses, with heart attack occurring in three out of the seven patients who died within the first year. Although a total of seven patients had different morbidities, none of them underwent reoperation. Infection was diagnosed as patients with noted surgical site discharge-. Most of these infections occurred within six weeks of surgery.

Compression hip screw fixation was the most common surgical method undertaken (Table II.) Patients who were surgically treated more than or less than two weeks after the injury were about the same at - 50% each. Table IV shows the total number of patients with assisted ambulation before the injury occurred. In reference to Figure 1, the total number of dependent ambulators is highest upon operation at six weeks but decreases steadily over time.

As shown in Figure 2, the Harris Hip Score was either good or excellent prior to injury but as soon as the surgery was performed, the Harris Hip Score was noted to improve from poor to fair within 12 months. Using the Kaplan Meier curve (Figure 3), it is noted that there is a 80% probability that a patient survives after 11 months (Relative SD = 0.045) at 95% confidence interval as shown in Table IVTable IV.

Using the log rank test, it can be noted that regardless of the type of surgery, presence of comorbities, delay in surgery and preoperative ambulation, the overall survival remained 11 months at 80% probability. None of these parameters are noted to be significant in changing the survival probability after the operation as shown in Table V. Discrimination analysis is used to determine the significant parts of the Harris Hip Score affected by the different patient factors. Based on Table VI, it can be noted that the parts of Harris Hip Score on sitting (p <0.05) and deformity (p< 0.05) were significantly affected by the type of surgery performed. Table VII shows the significant effect of delay in surgery in the distance travelled (p<0.05) and the range of movement (p<0.05) of patients after 12 months. Comorbidities were seen to significantly affect the amount of pain (p<0.05) and deformities (p<0.05) in Harris Hip Score at 12 months, while pre-operative level of ambulation affected the distance travelled and the ROMs after surgery.

Using multiple regression analysis, the parts of the Harris Hip Score which significantly affected the overall recovery (change from preoperative Harris Hip Score) were pain, support and distance travelled. Among these three factors, it can be noted that pain more than support more than distance travelled had greater degree of contribution to the overall recovery as can be seen by the greatest amount of coefficient in Harris Hip Score from preinjury to after 12 months and ambulation at 12 months.

Based on table above, it can be seen that the type of surgery significantly affected the pain (p=0.038) subitem in Harris Hip Score at 12 months. Conversely, the degree of comorbidity significantly affected pain (p=0.023) and deformity (p=0.023). Delay was noted to have a significant effect on the degree of change or recovery of function (p= 0.022). As expected, the pre-operative ambulation status significantly affected the post operative ambulation at 12 months (p<0.05).

Table VIII

## Discussion

Based on the results of this study, the most common patients with hip fractures undergoing surgery were women (81%). This is consistent with previous studies showing that hip fractures occur more frequently in the elderly female population, the probable reason being that post-menopausal women are at at greater risk for osteoporosis than men of the same age. Most of the patients in this age group, according to previous studies, showed poor functional status postinjury. Surgery in itself is another form of trauma, and thus, operating in this group to enable them to return to their preoperative functional level remains controversial.

We observed, however, in this study that regardless of the type of surgery, presence of comorbidities, delay in surgery or pre-ambulation status, the overall survivorship at 11 months remained high at 80%. At 6 months of surgery, the survivorship was as high as 90%. This means that even in the extremes of ages, as was reported in previous studies, surgery remains a good treatment option for better quality of life in the patients’ remaining years.

Despite the good overall survivorship, our results also showed that the change in independence of ambulation before the injury and after the surgery significantly decreased from 80 to 50% (p = 0.015). Having another traumatic insult through the surgery takes its toll on octogenarians in that most of them were not able to return to pre-injury functional status, thereby becoming functionally dependent throughout the remainder of their lives.

The Harris Hip Score, which has many sub-items, takes into account pain, limitation of function, and deformity in assessing post-surgery outcomes. Although this scoring system is used in previous studies in patients who underwent hip replacement, the researcher found it as informative and helpful in assessing the impact of surgery in extremes of ages as used in several studies. In discrimination analysis of the Harris Hip Score in relation to the premorbid factors, significant differences were noted in the Score. Both Spearman correlation statistic and discrimination analysis agree that the type of surgery and the presence of more than two comorbidities affect the degree of pain and deformity at 12 months. Octogenarians who underwent surgery, especially those with more than two comorbidities would have greater pain and deformity after 12 months. Delay in surgery of more than two weeks post-injury directly correlated to the distance the patient would be able to walk at 12 months post-op. Also, those patients with difficulty in ambulation without assistive devices, would predictably have difficulty walking at 12 months.

The overall functional recovery in terms of the difference in Harris Hip Score pre-injury and at 12 months post-surgery was reflected by the pain, use of supportive devices and distance travelled in decreasing degree of importance. This meant that to predict the part of Harris Hip Score that affected octogenarians at one year, one may only need to ask about patient’s pain, use of assistive devices or ability to travel distances.

The study is limited by the difference in levels of experience of the surgeons performing the surgeries. It must be noted that the patients’ mental status were not assessed, as this may greatly affect the post-operative mental function of the patients, which can directly affect the overall outcome. The dropout rate is acceptable as it is below 20%. Transportation expenses and difficulties of patients living in distant areas are problems which cause most of them to be lost to followup. The small number of patients also limits the power of this study.

One of the recommendations of this study is to have the follow-up duration be extended to more than a year to be able to know the survivorship for longer periods of time. Another is to include the assessment of mental function and impairments in patients, which can be a confounding factor that can alter results in terms of dependence in ambulation and the Harris Hip Score. The mental function can also be examined as a variable in future studies. To further limit variables, we also recommend that as far as possible, a single surgeon or surgeons of equivalent skills and competency perform the operation, using the same surgical approach for all hip surgeries.

**Table I: Demographics of Patients operated (N= 43) Harris Hip Score:**
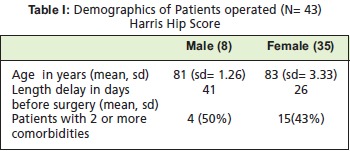


**Table II: Type of surgery - and its frequecy:**
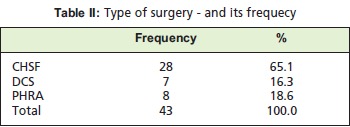


**Table III: Delay from time of injury -:**
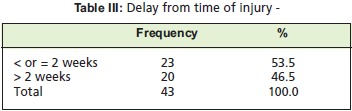


**Table IV: Preinjury ambulation. (Dependent ambulation = ambulation with crutches, walker or wheelchair):**
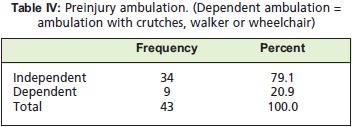


**Table V: Estimated survival time in months with 90 % probability:**
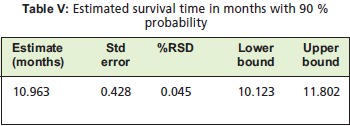


**Table VI: p values of different factors of patient which may affect survival curve. (p value < 0.05 is significant):**
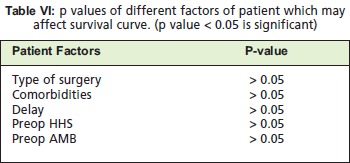


**Table VII: Significant items in the Harris Hip Score - in the overall - functional recovery (change in Harris Hip Score after 1 year):**
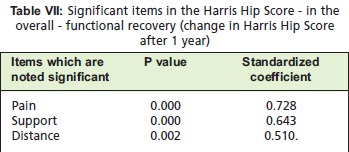


**Table VIII: Different pre-operative factors with significant effect on the functional outcome, change in Harris hip score from preinjury to after 12 months and ambulation at 12 months:**
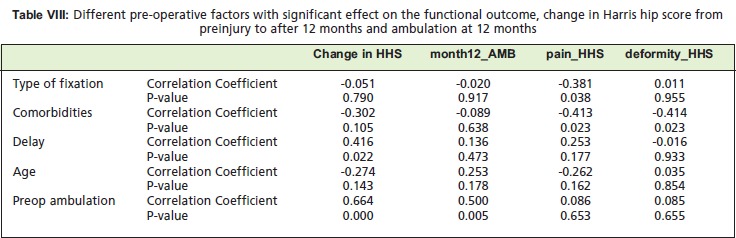


**Figure I: Total number of patients with assisted ambulation (blue) and without ambulation (green) at different periods of follow up.**
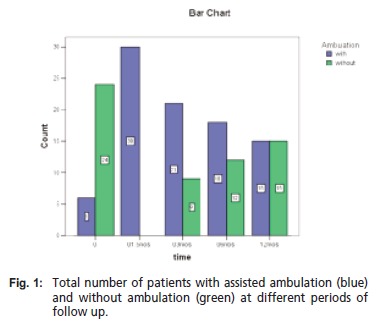


**Figure II: Harris Hip score. ( 90-100 for excellent, 80-90 good, 70-79 fair, 60-69 poor, and below 60 fail) vs time (months) post surgery.**
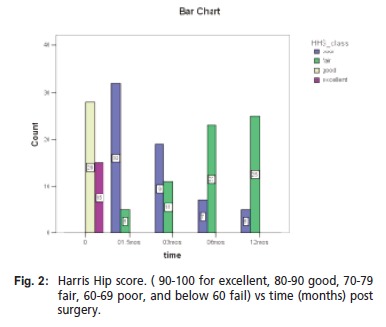


**Figure III: Survival vs time (in months) post surgery.**
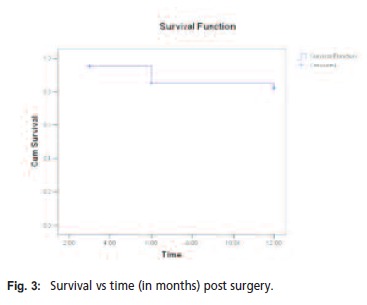


## Conclusion

The overall survival rate of octogenarians undergoing hip fracture surgery is 80% at 11 months. Survivorship at this rate is much acceptable and a good reason to convince patients and their families to undergo surgery despite the extreme of age. Because prolonged immobility and recumbence will cause increased morbidity mortality risk to the patient, surgery still proves to be beneficial. On the other hand, in terms of outcomes measured, the presence of two or more co-morbidities will cause greater pain and hip deformity at 12 months post-surgery, making the case for greater vigilance in doing surgery in these cases. Octogenarians using assistive devices pre-injury will have greater difficulty in community ambulation at 12 months. Delaying surgery more than two weeks will affect the resulting range of movement and ability to travel greater distances at two months, justifying early intervention if surgery is contemplated.
